# Unravelling the Glasgow effect: The relationship between accumulative bio- psychosocial stress, stress reactivity and Scotland's health problems

**DOI:** 10.1016/j.pmedr.2016.08.004

**Published:** 2016-08-03

**Authors:** Joe Cowley, John Kiely, Dave Collins

**Affiliations:** Institute of Coaching and Performance, School of Sport and Wellbeing, University of Central Lancashire, United Kingdom

**Keywords:** Scottish effect, Glasgow effect, Biopsychosocial stress, Physical activity

## Abstract

To date, multiple hypotheses have been proposed for the Scottish effect and, more specifically, Glasgow's high mortality rate and the associated Glasgow effect. Previous authors have highlighted the improbability of a single factor as responsible for this effect with seventeen possible hypotheses presented. These have ranged from socio-economic factors, lifestyle and cultural factors such as sectarianism, and political and economic factors. Although these may all be contributory factors to this paradox, the underpinning reasons for the observed effect remain relatively unexplained. In this paper, we suggest that the compounding effect of a unique blend of accumulating life stressors may predispose Scots, and particularly socially-disadvantaged Glaswegians, to a wide-range of health disorders. In short, a confluence of social, environmental, attitudinal and cultural stressors perhaps combine to negatively influence biological health. Future directions should consider the stress remediating role of physical activity, and the problems presented by barriers to participation in physical activity and exercise during key transitional stages of life.

## Highlights

• Evidence explaining the Scotland and associated Glasgow effects remains elusive.
• We suggest that accumulating life stressors predispose Scots to a wide-range of health disorders.
• Physical Activity (PA) and stress are bidirectional, we highlight the remediating role of PA.
• Future work should focus on barriers and facilitators of PA.
• Overcoming barriers to participation in PA and exercise at transitional stages of life is crucial.

## 1 Introduction

Since the 1950′s, Scottish life expectancy has improved more slowly than in other comparably wealthy nations. Furthermore, 43% of adults are classified as chronically ill or disabled and, despite medical advances, all-cause mortality in younger age categories (15–44 years) remains non-declining at a rate of 142.4 deaths per 100,000 (Bromley and Shelton, 2010). In addition, current suicide rates are 50% higher than in 1968 (Whyte and Ajetunmobi, 2010), obesity is on the rise, and general levels of physical activity are diminishing (Scottish Government and Convention of Scottish Local Authorities, 2010). As a consequence of this poor health record, and mortality rates persistently higher than European averages, Scotland has been dubbed ‘the sick man of Europe’, and the phenomenon of these unexplained health disparities labelled the ‘Scottish effect’ (Bromley and Shelton, 2010, Shelton, 2009, Whyte and Ajetunmobi, 2010).

Interestingly, within Scotland, further divergent health disparities are apparent. The West of Scotland, and more specifically the Greater Glasgow area, demonstrate particularly pronounced levels of premature mortality and an increased incidence of negative health outcomes and behaviours appear disproportionately common (Whyte and Ajetunmobi, 2010). Despite multiple suggested explanations for the Scottish effect, however, the phenomenon remains poorly understood and the underpinning drivers of the Scottish and Glasgow effects remain unclear. No single contributing factor appears to explain why Scotland, and Glasgow in particular, have different mortality patterns than other UK cities suffering comparable deprivation (McCartney et al., 2011). Indeed, it has recently been suggested that, whilst behavioural, socioeconomic and physiological factors certainly contribute to the Scottish effect, that psycho-emotional distress may exert an even greater contributing influence (McCartney et al., 2011, McCartney et al., 2015, Stults-Kolehmainen and Sinha, 2014).

Accordingly, within this article we explore the potential drivers of the Scottish effect through the lens of an updated 21st century understanding of the impact, on multiple dimensions of health, of excessively accumulating life stress. We suggest that the Scottish effect, and its nested sub-effects, can be explained through the convergence of multiple stress-promoting phenomena all conspiring to expose the Scottish population to unusually high levels of accumulating life stressors. Finally, we suggest that this re-framing of the Scottish effect underlines a crucial, if sometimes overlooked, argument for physical activity (PA) promotion amongst populations exposed to high levels of accumulating life stress: in short, systematic exploitation of the stress moderating benefits of regular PA.

## 2 Methodology

The electronic databases: *Medline, Embase, Cochrane reviews, Omnifile, ABI inform* and *Google scholar*, were searched for UK and worldwide academic literature published between 1985 and 2016, using the key search terms: Scotland effect; Glasgow effect; stress; health inequalities; Scottish and Glasgow health. Further resources were sought through associated public health journals, and from grey literature such as Government reviews, Glasgow Centre for population health data, and national evaluations. Although the search strategy employed a systematic approach, the breadth of this literature entailed that a full systematic review, as outlined by the Cochrane Collaboration, was not feasible.

## 3 Scottish, Glasgow and East Glasgow effects: Nested health effects illustrating the consequences of accumulative multi-source stress

In the late 1980's a UK-wide report highlighted that premature mortality in Scotland was more severe than in England or Wales (Carstairs and Morris, 1989, Walsh et al., 2010, Whynes, 2009a, Whynes, 2009b). Although these higher mortality rates were initially attributed to higher levels of deprivation, more recent investigations suggested that these health inequalities were not fully explained by deprivation levels alone. This phenomenon, of elevated mortality levels without a clear origin, was subsequently labelled ‘The Scottish Effect’ (Carstairs and Morris, 1989, Walsh et al., 2010, Whynes, 2009a, Whynes, 2009b). Although socio-economic status, cultural factors and other health-related risk factors all undoubtedly contribute to this phenomenon, an explanation as to why these health inequalities are so pronounced, remains elusive (Gray, 2008, McCartney et al., 2011).

As stated in the introduction, this effect seems even more pronounced in certain areas. Like many British cities, Glasgow, Scotland's largest city, underwent severe de-industrialisation in the mid 1980's, representing the fastest industrial decline within the United Kingdom (MacInnes, 1994). This de-industrialisation was accompanied by increasing deprivation and some of the worst premature mortality rates in Europe.

More recently, however, Glasgow has experienced rapid re-industrialisation, and growing prosperity. Nevertheless, as illustrated by recent Medical Research Council findings, Glasgow's health profile remains inferior to the rest of the UK (Gray et al., 2012). This circumstance suggests that the recent prosperity shift has not been reflected in a proportionally increasing health status (Hanlon et al., 2006) and that higher mortality rates are not explained by socio-economic deprivation alone (Hanlon et al., 2006, Walsh et al., 2010). Indeed, on analyzing data from the recent Scottish health survey, a Scottish Government report suggested living in Glasgow was independently associated with poorer health outcomes and increased all-cause mortality (Landy et al., 2010). This phenomenon, a further health disparity nested within the Scottish effect, has been termed ‘The Glasgow Effect’ (Gray, 2008, Walsh et al., 2010). The evidence linking low socio-economic status with elevated stress markers illustrates a clear link to a range of illnesses abundant in the West of Scotland (McEwen, 2008). Additionally, elevated levels of conditions related to anxiety, myocardial infarction, obesity and high GHQ scores (signifying potential psychiatric disorders) have been shown to be prevalent regardless of socioeconomic status (Landy et al., 2010).

In fact, other studies seem to suggest that even greater challenges exist. Within the Greater Glasgow area, there appears a further nested disparity. For example, males living in Bridgeton and Dennistoun have a life expectancy fifteen years lower than fellow Glaswegians residing, less than 5 miles away, in the more affluent Anniesland and Bearsden areas (Glasgow City Council Regeneration Team, 2011, Hanlon et al., 2006). Such inequalities are preserved across genders, with all-cause mortality rates ranging from 428 and 454 deaths per 100,000 for Anniesland and Bearsden, to 965 per 100,000 for Bridgeton and Dennistoun (Hanlon et al., 2006, National Records of Scotland, 2012). Notably, males from Dennistoun have only a 53% chance of reaching their 65th birthday (National Records of Scotland, 2012). Accordingly, some communities within the Greater Glasgow area exhibit the highest national levels of all-cause mortality, whilst others demonstrate the lowest.

In an international context, in the USA, residents of the Appalachian counties display a similar pattern of excess mortality to that of Scotland, with CHD death rates exceeding the national average by between 15 and 21%. It is hypothesized that these disparities occur as a result of a combination of socioeconomic stressors and cultural barriers to accessing the health care system (Barker et al., 2010, Center for Disease Control and Prevention- CDC, 2002).

### 1.3.1 The search for an explanation

To date multiple hypotheses have been offered explaining the Scottish and Glasgow effects. A recent report identified seventeen possible hypotheses —ranging from socio-economic; cultural; political; genetic and climatic factors— before concluding:

*There was clearly a large number of outstanding deductive hypotheses which could be investigated for their potential causal role in generating the mortality pattern in Scotland (Glasgow in particular). There remains room, however, for further inductive work into the divergence of Scottish mortality* *from the rest of Europe around 1950* (McCartney et al., 2011).

Against this backdrop, we feel that the cumulative effect of multiple stress-inducing factors, and their interactions, may offer the most parsimonious explanation for this phenomenon.

### 1.3.2 The evolution of the science of stress

As noted by Wheaton (1994), a clear understanding of the ‘stress’ phenomenon is inhibited by the many interpretations and connotations associated with the term, as well as its common indiscriminate usage within contemporary culture (Wheaton, 1994). From an academic perspective, stress was historically defined from within discipline-specific constraints. Thus biologically-oriented researchers historically neglected the stress modulating influences of psycho-emotional factors, such as: perception, emotional appraisal, psycho-social coping mechanisms, anticipation and learning (Ganzel et al., 2010). An omission recognized by the most prominent of the early stress researchers, Hans Selye, when noting, late in life, that he had long-envisioned stress as a “purely physiological and medical phenomenon” (Selye, 1983). In similar vein, more psychological and sociological-oriented academics failed to fully appreciate the influence of psycho-emotional stress on the full spectrum of physiological dimensions of health (Ganzel et al., 2010, Kopp and Réthelyi, 2004).

In recent years, however, aided by the rapid evolution of neuro-imaging technology, this historical disconnect —between biological and psycho-emotional stress— has largely been resolved (Ganzel et al., 2010). Contemporary stress paradigms now depict stress as an integrated biopsychosocial phenomenon whereby, regardless of the genesis of the stressor, the subsequent defense responses mobilized inevitably exert wide-ranging neurological, endocrinological, physiological and hence, inevitably, impose general health consequences (Ganzel et al., 2010, Marmot, 2000, McEwen, 2008).

Accordingly, within public health domains, a rapidly accumulating literature documents the role of excessively accumulating life ‘stress’ in driving a broad diversity of negative health outcomes: outcomes encompassing multiple —psychological, neurological, cognitive, social and emotional— dimensions of human function, across every segment of the lifespan (Carlsson et al., 2014, Everson-Rose and Lewis, 2005, Hackney, 2006).

As illustration, traumatic adversity —in childhood, middle-age or old-age— has been extensively linked to detrimental physical, mental and emotional health outcomes (Appleton et al., 2012). Similarly, it is well established that exposure to incidents of extreme stress and/or chronically-applied stressful circumstance in early life, such as growing up in impoverished or disadvantaged conditions, typically increases the likelihood of negative future health outcomes (Appleton et al., 2012). Furthermore, recent evidence, illustrates that regular and repetitive exposure to seemingly minor everyday stressors —which Wheaton (1994) termed ‘daily hassles’—also exerts a toll on psycho-physiological health (Aldwin et al., 2014). This expanding body of evidence suggests that the chronic exposure to both major and minor stressors — even those as apparently inconsequential as frequent arguments with spouses, neighbours or simply watching others in stressful situations— progressively accumulate and inevitably negatively impact multiple dimensions of health (Lippold et al., 2014, Stawski et al., 2013, Zoccola et al., 2014). In evaluating this suggestion, we firstly consider the mechanisms through which stress influences general health.

### 1.3.3 What is stress; what causes stress?

Fundamentally, the stress response is launched by a change in emotional state prompted by perceived ‘threat (Ganzel et al., 2010).

This change in emotional state instigates alterations in the individual's neuro-chemical profile which, in turn, drives an array of biological, emotional and psychological consequences evolutionarily designed to prepare the organism for evasive action (McEwen, 2005, McEwen, 2008). In essence, the stress response is an evolutionary embedded survival strategy, designed to prepare and protect the organism from imposed dangers. Accordingly, when life circumstances trigger an emotional response, such as anxiety or fear, neuro-chemicals are released to better enable the organism to remediate the source of emotional discomfort. Most famously, stress increases secretion of the neuro-transmitter cortisol. Like other neurochemicals and hormones, cortisol exerts an array of context-dependent influences on behaviour: priming cognition, sharpening focus, increasing energy availability and damping pain sensations (Wirth, 2014). Elevated cortisol levels thus ready the brain and body for the evasive actions necessary to counter the source of perceived threat. Crucially, however, excessive exposure to the corrosive effects of cortisol, and downstream neural correlates, exacerbates ‘wear and tear’ of the neural circuitry responsible for modulating stress reactivity, degrading the neural processing centres most intimately involved in initiating and terminating stress responses and, in so doing, heightening future stress reactivity and further exacerbating wear and tear.

This cycle of increasing reactivity and vulnerability to future stress exposures leads, ultimately, to accelerating functional decline. Consequently, excessive exposure to historical stress subjects individuals to the dual threat of heightened stress reactivity and a diminished capacity to efficiently regulate stress responses, thereby raising susceptibility to future stress induced ‘damage’(McEwen, 2005, McEwen, 2008, Wirth, 2014).

This downward spiral is a particularly powerful concomitant of modern 21st-century lifestyle. The stress response evolved to cope with the survival challenges encountered by our Paleolithic ancestors. In contemporary 21st century contexts, however, where short-term physical threats are rare but long-term psycho-emotional pressures are many, prolonged activation of the stress response exposes our neuro-biological system to the corrosive effects of prolonged exposure to the stress response's chemical cascade: long-term exposures which act to progressively degrade resilience to future stressors. Supporting this perspective is the rapidly expanding evidence illustrating excessive stress reactivity as a main contributor to many common 21st century ailments (Juster et al., 2010, McEwen and Sapolsky, 1995, McEwen, 1998).

### 1.3.4 The individual experience – It's the stress response, not the stressor

A confounding barrier to understanding ‘stress’ is that the stress response may be launched in response to any life event. Crucially, it is important to note that the magnitude of the stress response is not solely dependent upon the magnitude of the stressor, but is heavily modulated by the individual's sensitivity to the particular stress (Nabi et al., 2013). Furthermore, the health and behavioural consequences of accumulating stress are dependent on individual stress resilience. Individual sensitivity, in turn, is shaped by a broad coalition of innate genetic and pre-dispositional factors, and is similarly heavily influenced by cultural and attitudinal factors forged by individual life history. Accordingly, our stress reactivity is shaped by a blend of genetic, behavioural (coping and health habits), historical (developmental experiences, prior stress exposures) factors, early life experiences and cultural attitudes (Juster et al., 2010, McEwen and Sapolsky, 1995).

Although high levels of stress, especially in early life, can serve to increase an individual's stress-resilience, if this stress is excessive or over-whelming, then early life stress can predispose the individual to a life-long vulnerability to future stressors (Juster et al., 2010). In simple terms, a lack of perceived self-efficacy in ability to cope with an imposed stressor entails that the negative impacts of that stressor are magnified. As a consequence, many of those exposed to high levels of trauma in early life, exhibit a lifelong predisposition to disproportionately excessive reactivity to imposed stress (Appleton et al., 2012). This reactivity exposes individuals to an increased likelihood of future stress-induced wear and tear, increased susceptibility to stress-related illnesses, and subsequently accelerating health decline. The health consequences of exposure to an excessively activated stress response are manifest in the increasing incidence of a host of 21st-century ailments such as obesity, cardiovascular disease and psychiatric disorders (Juster et al., 2010).

### 1.3.5 Biopsychosocial stress and the Scottish effect: A storm of Scottish stressors

It seems apparent that low socio-economic status is an inherent pre-disposing factor to stress, accounting for a large proportion of the stress burden imposed on individuals. Nevertheless, there remain certain enigmatic stress modulating characteristics of life in Scotland, and specifically in Glasgow, which further add to the health-reducing toll of accumulative stress. For example, resistance to a healthy lifestyle may be endemic within Scottish culture (O′Brien et al., 2009). Certainly there appears a cultural disinclination, particularly amongst males, to discuss, or engage in, positive health behaviours (Courtenay, 2000). As illustration, it has been suggested by O′Brien, Hunt, and Hart (2009, p376) that Glaswegian males were ‘*motivated to align themselves to the kind of masculinity that was valorised by their peers in order to avoid feeling ostracised’,* portraying an image of masculinity characterised by the adoption of risky health behaviours, such as competitive drinking and unhealthy diets, and an inherent resistance to the adoption of ‘good’ health practices (Sloan et al., 2009). Recent studies have similarly noted a ‘macho’ approach to health behaviours in Scottish men. In particular, many men considered discussion, or practice of good health behaviours, such as healthy dietary and exercise habits as feminine; whilst negative health behaviours, such as binge drinking were perceived as manly (Courtenay, 2000, O'Brien et al., 2009, Sloan et al., 2009).

The suggestion that health inequalities in Scotland and more specifically Glasgow, are directly dependent upon deprivation offers a partial truth, but not explanatory closure. After controlling for deprivation, it seems apparent that the mortality disadvantage embedded within this phenomenon is worsening. Indeed, Scotland's relative ranking in relation to younger working age mortality, compared to other European countries, has progressively worsened for both sexes over the last 55 years (Whyte and Ajetunmobi, 2010). Furthermore, many health-related indicators, such as psychological morbidity, death from all cancers, chronic liver disease, and inadequate dietary intake of fruit and vegetables seems pervasive across socio-economic Glaswegian groups (Whyte and Ajetunmobi, 2010).

Apart from socio-economic status, deprivation and social inequality, other biopsychosocial stress-inducing factors have also been suggested as adding to the overall stress burden potentially driving the Glasgow effect: such as, for example, the physical and the climatic environment (McCartney et al., 2011, Reid, 2009), and the social legacy of immigration whereby descendants from Irish immigrants in Glasgow showed an increase in premature mortality, even subsequent to controlling for established risk factors (Abbotts et al., 1998). Accordingly it seems clear there is no simple, single causative factor ultimately responsible for the Scottish, and nested Glasgow effects (Reid, 2009).

#### 1.3.5.1 Unrelenting biopsychosocial stress and social health

Our suggestion that apparently separate influencing factors can be understood within a unifying explanatory rubric of cumulative multi-source life stress, unites these factors within a single conceptual model. The biopsychosocial impact of multi-source stressors results in greater accumulative life stress, heightening the risk of stress-related negative health outcomes. The burden of this chronic stress is accompanied by culturally promoted changes in personal behaviours: such as increased incidence of smoking, disordered eating and drinking. Stress-inducing lifestyle behaviours, in turn, drive other stress-elevating conditions, such as poor quality sleep, increasing body mass index, reducing energy levels and reduced tendencies to engage in health-promoting physical activity behaviours (McEwen, 2008). In short, these factors interact in a downward spiral, adding momentum to an insidious vicious cycle of self-perpetuating stress whilst, simultaneously, over-activation of the stress response erodes stress resilience.

## 4 Simple solutions alleviating a complex problem?

Contextualising the Scottish effect as the insidious accumulation of relatively minor, but pervasive and persistent, stressors provides a conceptual model illuminating a previously incompletely explained phenomenon. Further, a shift in how we conceptualise the problem re-emphasises the remedial potential value of a certain simple, straight-forward and cost-effective strategies: physical activity (PA).

The rationale for promoting vigorous PA has traditionally focussed upon the well-established benefits to physical health (Cooper and Hancock, 2011). However, in recent years, multiple strands of research have emerged demonstrating a positive relationship between PA and a range of emotional, cognitive and mental health capacities (Martikainen et al., 2013). For example, PA has been shown to exert large to moderate positive effects on depression, anxiety-related disorders, and ADHD (Dinas et al., 2011, Hoza et al., 2014). Similarly, vigorous exercise has been demonstrated to enhance various dimensions of emotional regulation such as mood, self-esteem, and impulse control (Chaddock et al., 2011, Davis et al., 2011, Voss et al., 2011). In addition, and perhaps critically, physical exercise serves to increase emotional resilience against stressors yet to be experienced (Smith, 2013). These potential benefits are well supported by available research. Cross-sectional studies illustrate that regular exercise is associated with enhanced wellbeing, exerting positive effects on mood and anxiety symptoms (Goodwin, 2003). Further, growing evidence reveals positive relationships between PA, physical fitness, selected measures of cognitive function and academic performance (Biddle et al., 2000).

Initiatives elsewhere, focusing on positively influencing lifestyle change, have demonstrated significant improvements in health factors. By way of example, the Finnish North Karelia project —a community outreach programme emphasising lifestyle, PA and dietary factors— demonstrated significant reductions in cardiovascular disease (Puska, 2002). As a consequence, mortality from Coronary Heart Disease declined by 73%, while in the same period the Finish national average declined by only 65% (Puska, 2002). Furthermore self-reported levels of PA demonstrated a positive independent dose relationship with lowered mortality rates (Hu et al., 2005).

In the Appalachian areas of the USA, physical inactivity as a result of deindustrialisation has been evidenced as a driving factor in the poor health status of the region (Hortz et al., 2009). The magnitude of the problem of low PA in Appalachia has merited the attention of several investigations looking at the barriers and facilitators to PA in children and adolescents aged 8–17 years old (Swanson et al., 2013).

### 1.4.1 The case for PA in solving health problems

Jeremy Morris, the former CMO for the UK, branded PA and exercise as “the best buy in public health for the West”(Morris, 1994). The burden of physical inactivity and its associated factors have been highlighted by Wanless (2004), reporting that inactivity costs the NHS £8.2 billion annually, whilst the increasing costs of health care, hospitalisation, medication and operative procedures for CHD costs the NHS a further £10 billion annually (Wanless, 2004). In a Scottish Context, recently the Chief Medical Officer for Scotland highlighted that 9% of our population die as a result of physical inactivity, with the burden of inactivity in Scotland costing over £800 million each year (Calderwood, 2016). Epidemiological evidence has similarly highlighted the importance of promoting PA.

By way of example, it is estimated that physical inactivity is associated with 6–10% of the major non-communicable diseases of coronary heart disease, type 2 diabetes, and breast and colon cancers. Additionally, in the United Kingdom, inactivity is considered a cause of 9% of all premature mortality, equating to more than 5·3 of the 57 million deaths in 2008 (Lee et al., 2012).

Of additional relevance, PA undertaken within natural environments —parks, woodlands, trails has been evidenced to provide an extra stress-reducing effect: potentially hinting at the benefits of exposure to natural scenery and context more aligned with our shared evolutionary heritage (Aspinall et al., 2013, Tyrväinen et al., 2014).

## 5 Conclusion

There is a profound mismatch between the historical contexts, within which we evolved, and 21st century life as experienced in first World countries. The stress response, which originally evolved to protect us from immediate short-term physical danger, is now habitually activated in response to commonplace, everyday events —such as financial worries, work-related pressure or perceived low social status. Thereby placing a heavy burden on human neuro-physiological defense mechanisms.

Chronic activation of the stress response, in response to minor events perceived to be threatening —as opposed to truly threatening— events, exposes the biological system to the corrosive consequences of repeated over-expression of the bio-chemistry originally designed to cope with short-term ‘threats’. In essence, it is not the magnitude of applied stressors driving many health problems, but the negative consequences of persistent exposure to toxic levels of elevated stress hormones (Ganzel et al., 2010).

Negative health behaviours are a key driver of poor health. These behaviours may be culturally embedded as deep rooted beliefs, attitudes and perceptions, but ultimately all factors conspire to add to the cumulative stress burden to which individuals within that culture are exposed (Sheridan et al., 2013).

Within this paper we have employed the Scottish effect as a lens through which to contextualize the root cause of many 21st century health problems: accumulating and un-remediated multi-source life stress. However, this problem is certainly not exclusive to Scotland, and in fact is a pervasively growing first World problem. Nevertheless, the Scottish population, for the range of reasons highlighted here, does seem particularly vulnerable to the spectrum of modern stress-related health issues. Given that Scotland has a deep rooted culture of obesity and low PA levels, it is evident that these issues need to be addressed in order to become a more active and healthy population. Furthermore, it seems warranted that the deep rooted cultural attitudes and beliefs, which contribute to shaping behavioural influences, be tackled within the context of health promotion initiatives.

Future research directions could profitably investigate cultural aspects of machismo, such as poor attitude towards exercise and PA, and perceived increases in male stature that accompanies bouts of heavy drinking, and other risky behaviours. Given that the cultural embodiment of these factors may be important when analyzing the paradoxical Scotland effect, future research should address the issue of why so many males continue to engage in pseudo-rituals of masculinity that are likely to be harmful to health (O'Brien et al., 2009). The promotion of healthy behaviours, such as a rejection of binge drinking together with implementation of minimum alcohol pricing, has been a recent focus of the Scottish Government. However males, particularly those already drinking at higher risk levels, appear less likely to support this policy (ScotCen Social Research, 2013).

Despite the entwined social, cultural, geographical, genetic and personal history underpinning our vulnerabilities and resilience's to accumulating life stress, and despite the expansive range of commonly suggested stress-remediating strategies, PA offers perhaps the most flexible, cheapest, readily accessible and logistically feasible evidence-led means of countering the negative stress-related consequences of our otherwise privileged position as citizens of first World 21st century life. The bi-directional relationship between PA and stress resilience further underscores the criticality of embedding early, life-long PA habits, especially in populations at heightened risk of stress-related health impediments. Physical Education philosophy and the appropriate training of Physical Educators may subsequently play a crucial role in fostering the positive culture change, associated with ensuring sustainable PA during the important transitional phases of adolescence.
